# Innovation and reform: China's 14th Five-Year Plan unfolds

**DOI:** 10.1093/nsr/nwaa294

**Published:** 2020-01-15

**Authors:** Mu-ming Poo

The Chinese government has recently announced a comprehensive proposal of the 14th Five-Year Plan for the next phase of development in all sectors of Chinese society. The Plan is formulated within the context of China's long-term goal of building a moderately prosperous (‘xiaokang’) society by 2035. Importantly, the Plan includes detailed guidelines for the development of science and technology (S&T), with the resounding themes of innovation and reform. In particular, it clearly designated the focused research areas and goals for system reform in Chinese research institutions and related industries.

Major S&T projects (known as ‘Innovation 2030’) will be initiated in the frontier areas of artificial intelligence, quantum information, integrated circuit, public health, brain science, breeding biotechnology, deep-earth and deep-sea research, and space technology. These projects will be facilitated by optimizing and establishing enabling facilities and mechanisms of resource sharing. In line with the focused areas, new national laboratories will be established, and existing state key laboratories will be reorganized. Much of the efforts will support the development of Beijing, Shanghai and Guangdong-Hong Kong-Macao regions into China's three major international centers for S&T innovation.

While innovation and reform are not new themes, the Plan stipulates several concrete steps. A notable new point is to support industry-led joint organizations for taking a leading role in major national S&T projects, which in the past were largely led by research institutions such as Chinese Academy of Sciences and research universities. Tax benefit will be granted to industry's investment in basic research to support industry as a source of innovation. Proper allocation of the personal credit for inventions will be enforced. Basic research innovation will be linked into industrial chains, pushing the high-end, smart and green development of traditional industries.

Successful drive for innovation needs substantial reform of research organization. According to several estimates, China now has an S&T workforce of more than 100 million, the largest in the world. The government's investment in S&T development now comprises 2.1% of GDP, approaching the level of developed countries. However, major innovation in many areas remains disproportionally low. As pointed out by President Xi Jinping in a recent meeting with scientists, the key is to improve the ecology of research environment for innovation. The Plan addresses this issue by a detailed list for reforms, including the mechanisms for cultivating first-rate innovative talents with an international view, the implementation of ‘chief scientist in charge’ system, the expansion of autonomy in decision making within the organization and management of major S&T projects, as well as the establishment of personnel evaluation systems that are based on innovative quality and productivity of the research.

Innovative minds are shaped by education and life's experiences. The Plan calls for supporting the development of high-level research universities and strengthening the education of basic research talents. While more funding to universities is expected, a more critical aspect appears to lie in the reform of Chinese education system, including not only universities but also primary and secondary schools. The curricula and teaching material need to be designed in a way that is not only to infuse existing knowledge but also to instill inquiry into the unknown, and the teaching style of the faculty needs to change from didactic toward inspirational. To meet the need for innovative talents, the reform of Chinese education system has become increasingly urgent.

The inception of the 14th Five-Year Plan occurs at a time that China is facing great challenges on both domestic and international fronts. Highlighted in the Plan is China's resolve to actively foster a sound international environment for the S&T development. In the post-COVID era, China will promote healthy and collaborative international relations, defend the UN-centered international system and order, and actively participate in international efforts to meet global challenges in S&T, such as global warming and the prevention and control of major infectious diseases. Future will testify that the next phase of China's development, as charted by the Plan, represents a critical moment in history not only for China but also for the entire world.

